# The longitudinal dimension of the ascending aorta: beyond diameter

**DOI:** 10.1186/s44348-026-00098-1

**Published:** 2026-10-20

**Authors:** Sung Min Ko

**Affiliations:** Department of Radiology, Wonju Severance Christian Hospital, Yonsei University Wonju College of Medicine, 20 Ilsan-Ro, Wonju, 26426 Korea

Maximal ascending aortic diameter remains the principal imaging measurement used to monitor thoracic aortic aneurysm and guide prophylactic intervention. This approach is practical, reproducible, and supported by outcome data, but it reduces a complex three-dimensional structure to a single cross-sectional measurement. Current guidelines therefore retain diameter as the central criterion while allowing lower thresholds in selected high-risk settings [1]. Nevertheless, many patients with acute type A aortic dissection (ATAAD) present with an ascending aortic diameter below the conventional 5.5-cm threshold [2]. Diameter captures an important component of risk, but it does not fully characterize the aortic phenotype that precedes dissection.

This limitation has increased interest in the longitudinal dimension of the ascending aorta. Prior studies have associated ascending aortic elongation with adverse aortic events and ATAAD, and predissection imaging studies have suggested that length and volume may provide information beyond diameter alone [3–5]. Biomechanically, this concept is plausible: the ascending aorta is exposed to both circumferential and longitudinal stresses, and increased length may reflect altered wall structure, stiffness, or axial loading not captured by maximal caliber [6]. The key question is what additional disease feature length captures and whether it improves risk assessment when combined with diameter.

In this issue of the Journal of Cardiovascular Imaging, Boudreaux et al. [7] compare thoracic aortic morphology among 50 controls, 61 patients with ascending aortic aneurysm, and 13 patients with computed tomography (CT) angiography before subsequent ATAAD. Ascending aortic length (AAL), defined as the centerline distance from the sinotubular junction to the origin of the brachiocephalic artery, was 6.38 ± 0.75 cm in controls, 9.03 ± 1.08 cm in the aneurysm group, and 10.73 ± 1.41 cm in the predissection group. AAL was significantly greater in the predissection group than in the aneurysm group (*P* < 0.001), and this difference persisted after adjustment for age, sex, and height. Thus, elongation was shared by both disease groups but was more pronounced before dissection.

Interpreting length in relation to maximal ascending aortic diameter (MaxD) further clarifies this pattern. MaxD was largest in the aneurysm group (4.96 ± 0.52 cm), intermediate in the predissection group (3.89 ± 0.60 cm), and smallest in controls (3.11 ± 0.30 cm). All predissection diameters were below 5.5 cm, with a maximum of 4.9 cm. The AAL/MaxD ratio was 2.06 ± 0.22 in controls, 1.83 ± 0.21 in the aneurysm group, and 2.80 ± 0.50 in the predissection group (all pairwise *P* < 0.001). The corresponding areas under the receiver operating characteristic curves were 0.94 (95% confidence interval, 0.87–0.99) for predissection versus control and 0.99 (0.97–1.00) for predissection versus aneurysm. Both absolute length and the ratio therefore identified group differences; the ratio describes disproportionate elongation rather than establishing prospective dissection risk.

This distinction supports a clinically relevant hypothesis. Aortic disease may not always progress along a single sequence from normal caliber to diffuse aneurysmal enlargement and then to dissection. In this study, the aneurysm cohort showed both elongation and more diffuse thoracic aortic dilatation, including the arch and descending aorta. In contrast, the predissection cohort showed marked elongation, only moderate ascending aortic dilatation, and relative preservation of the distal thoracic aorta. These findings are consistent with at least two remodeling patterns: a predominantly radial, diffuse aneurysmal phenotype and a more longitudinal, ascending-aorta-predominant phenotype. The study, however, cannot establish two distinct biological pathways or a temporal sequence. Rather, it offers an exploratory morphologic framework for risk stratification that requires longitudinal validation.

The AAL/MaxD ratio also requires statistical caution. A higher ratio can result from greater length, a smaller diameter, or both. Because the aneurysm group was selected using a diameter criterion, its separation from the predissection group partly reflects the denominator and cohort definitions. The cutoffs were derived and evaluated in the same dataset, with only 13 predissection cases among 151 patients undergoing ATAAD repair. Bootstrap confidence intervals quantify sampling uncertainty but do not establish external validity or validate the selected cutoffs. These results describe discrimination between selected groups, not the probability of future dissection in a surveillance population. Future studies should compare models containing AAL and MaxD as separate variables with models using their ratio, assess incremental value beyond clinical risk factors, and validate performance independently.

Equally important is measurement standardization. The authors clarify that all examinations performed at their institution were electrocardiographically gated, whereas gating status could not be confirmed for outside examinations, which constituted most predissection scans. This uncertainty should not be equated with confirmed non-gated acquisition. Motion, reconstruction parameters, and measurement conventions may affect aortic dimensions [8]. Lengths measured from the sinotubular junction should also be distinguished from those beginning at the annulus. Excellent interobserver agreement, including an intraclass correlation coefficient of 0.99 for AAL/MaxD in 72 paired assessments, supports measurement reproducibility within this study; consistency across protocols, software platforms, and centers remains to be established.

Body-size normalization is another unresolved issue. Height-indexed analyses and adjustment for age, sex, and height supported the principal findings, whereas body-surface-area-indexed results were not reported. Height-based indexing is a practical approach [9], but future studies should evaluate which absolute and indexed measures provide complementary information. Collecting weight, blood pressure, hypertension and smoking history, genetic background, and valve status would also help distinguish longitudinal remodeling associated with aortic disease from variation related to body size, aging, and other clinical factors.

The timing of imaging also matters. In the 13 paired examinations, AAL and MaxD increased by 23.3% and 22.9%, respectively, between predissection and at-dissection CT. The intervals ranged from 29 to 868 days, so these differences combine interval growth with changes occurring during dissection. The absence of a significant interval–change correlation in this small sample cannot separate those contributions. Because dissection itself alters aortic geometry [10], these observations should not be interpreted as measurements of acute elongation alone.

The most useful next step is a multicenter longitudinal study using a prespecified measurement protocol. Repeated imaging could determine whether longitudinal growth, radial growth, or discordance between the two carries the greatest risk. Recent work combining diameter, length, and their growth rates supports evaluation of this integrated approach [11]. Automated or semiautomated centerline analysis may facilitate implementation, alongside assessment of aortic volume, tortuosity, arch geometry, wall characteristics, and clinical risk factors. Such models should demonstrate reliable prediction and clinically useful improvement over existing assessment before influencing intervention decisions.

The study by Boudreaux et al. [7] provides a rationale for evaluating the longitudinal dimension of the ascending aorta without proposing a new surgical threshold. Its contribution is the identification of a predissection morphology characterized by greater absolute length and disproportionate elongation despite only moderate dilatation. If confirmed in larger, standardized cohorts, this multidimensional perspective may improve recognition of patients whose aortic geometry is incompletely characterized by diameter alone. For cardiovascular imaging, the opportunity is to describe how the aorta remodels along its length and determine whether that information improves risk assessment.

## Data Availability

No datasets were generated or analysed during the current study.
